# Chemosensitization of *Trypanosoma congolense* Strains Resistant to Isometamidium Chloride by Tetracyclines and Enrofloxacin

**DOI:** 10.1371/journal.pntd.0000828

**Published:** 2010-09-28

**Authors:** Vincent Delespaux, Hervé Sèna Vitouley, Tanguy Marcotty, Niko Speybroeck, Dirk Berkvens, Krisna Roy, Stanny Geerts, Peter Van den Bossche

**Affiliations:** 1 Animal Health Department, Institute of Tropical Medicine Antwerp, Antwerp, Belgium; 2 Centre International de Recherche – Développement sur l'Elevage en Zone Subhumide, Bobo-Dioulasso, Burkina Faso; 3 Pathology and Parasitology Department, Faculty of Veterinary Medicine, Chittagong Veterinary and Animal Sciences University, Khulshi, Chittagong, Bangladesh; 4 Department of Veterinary Tropical Diseases, Faculty of Veterinary Science, University of Pretoria, Onderstepoort, South Africa; University of Tokyo, Japan

## Abstract

**Background:**

Because of the development of resistance in trypanosomes to trypanocidal drugs, the livelihood of millions of livestock keepers in sub-Saharan Africa is threatened now more than ever. The existing compounds have become virtually useless and pharmaceutical companies are not keen on investing in the development of new trypanocides. We may have found a breakthrough in the treatment of resistant trypanosomal infections, through the combination of the trypanocide isometamidium chloride (ISM) with two affordable veterinary antibiotics.

**Methodology/Principal Findings:**

In a first experiment, groups of mice were inoculated with *Trypanosoma congolense* strains resistant to ISM and either left untreated or treated with (i) tetracycline, (ii) ISM or (iii) the combination of the antibiotic and the trypanocide. Survival analysis showed that there was a significant effect of treatment and resistance to treatment on the survival time. The groups treated with ISM (with or without antibiotic) survived significantly longer than the groups that were not treated with ISM (P<0.01). The group treated with the combination trypanocide/antibiotic survived significantly longer than the group treated with ISM (P<0.01). In a second experiment, groups of cattle were inoculated with the same resistant trypanosome strain and treated with (i) ISM, (ii) ISM associated with oxytetracycline or (iii) ISM associated with enrofloxacine. All animals treated with ISM became parasitaemic. In the groups treated with ISM-oxytetracycline and ISM-enrofloxacine, 50% of the animals were cured. Animals from the groups treated with a combination trypanocide/antibiotic presented a significantly longer prepatent period than animals treated with ISM (p<0.001). The impact of the disease on the haematocrit was low in all ISM treated groups. Yet, it was lower in the groups treated with the combination trypanocide/antibiotic (p<0.01).

**Conclusions/Significance:**

After optimization of the administration protocol, this new therapeutic combination could constitute a promising treatment for livestock infected with drug resistant *T. congolense*.

## Author Summary

African Animal Trypanosomiasis causes the death of 3 million head of cattle each year. The annual economic losses as a result of the disease are estimated to be 4.5 billion US dollars. Trypanosomes are transmitted by tsetse flies and can infect a wide range of hosts from wildlife to domestic animals. This study is dealing with *Trypanosoma congolense*, which is one of the very prevalent parasites affecting livestock of poor African rural communities, decreasing the milk and meat production but also reducing the fitness of cattle that is used as draught power. Infected animals can only be treated by three compounds, i.e., diminazene, isometamidium and ethidium. These three products have been in use for more than a half century and it is thus not surprising to observe treatment failures. In some areas, the trypanosomes circulating have developed resistance to the three drugs leaving the farmers with no further options. As pharmaceutical companies are not keen on investing efforts and money in the development of new veterinary drugs for this low-budget market, our idea was to render an old ineffective drug effective again by combining it with existing potentiating compounds that are available and affordable for the livestock keeper.

## Introduction

African Animal Trypanosomiasis affects about 10 million km^2^ of sub-Saharan Africa and is a primary cause of rural poverty and food insecurity as explicitly recognized by the African Union, FAO and others [1]. Tsetse and the disease they transmit will continue to be a considerable threat to livestock and rural development [2]. Over the years, a large arsenal of vector control tools has been developed but they are difficult to sustain at the smallholder level. Hence, the control of animal trypanosomiasis (mainly *Trypanosoma congolense*) and zoonotic Human African Trypanosomiasis (mainly *T. brucei rhodesiense*) in poor rural communities has and will continue to rely heavily on the use of trypanocidal drugs. However, the development of trypanocidal drug resistance in *T. congolense* was reported in 17 countries of sub-Saharan Africa [3] and is becoming a huge threat for the cattle breeders in many regions. On the Adamaoua plateau in Cameroon, for example, up to 100% of the tested trypanosome isolates were found resistant to isometamidium chloride (ISM) and to diminazene aceturate (DA) leaving farmers helpless [4]. Unfortunately, no new drug is expected to be available in the near future and resistance is spreading very rapidly. For example, a five fold increase in DA resistance within a seven years interval was observed in the Eastern Province of Zambia [5]. Hence, alternatives are urgently needed to circumvent trypanocidal drug resistance.

Reversal of drug resistance or chemosensitization was successfully achieved, among others, in yeast [6], *Plasmodium* [7], [8], cancer cells [9] and *Leishmania* [10]. Such strategies could bring a much needed relief to African livestock breeders if they could be implemented at a reasonable price by shortcutting the development of new compound, toxicity studies and long clinical trials.

Many bacterial secondary multidrug resistance transporters belonging to the two major families, i.e. the Major Facilitator Superfamily (MFS) and the Multi Antimicrobial Extrusion Family (MatE) are described as having affinity for ethidium bromide (Homidium) as well as for many different compounds such as plant alkaloids, noxious metabolic products (such as fatty acids or bile salts), organic solvents and diverse antibiotics [11]. At least eight representatives of those transporters families are present in the genome of *T. congolense*.

**Figure 1:**
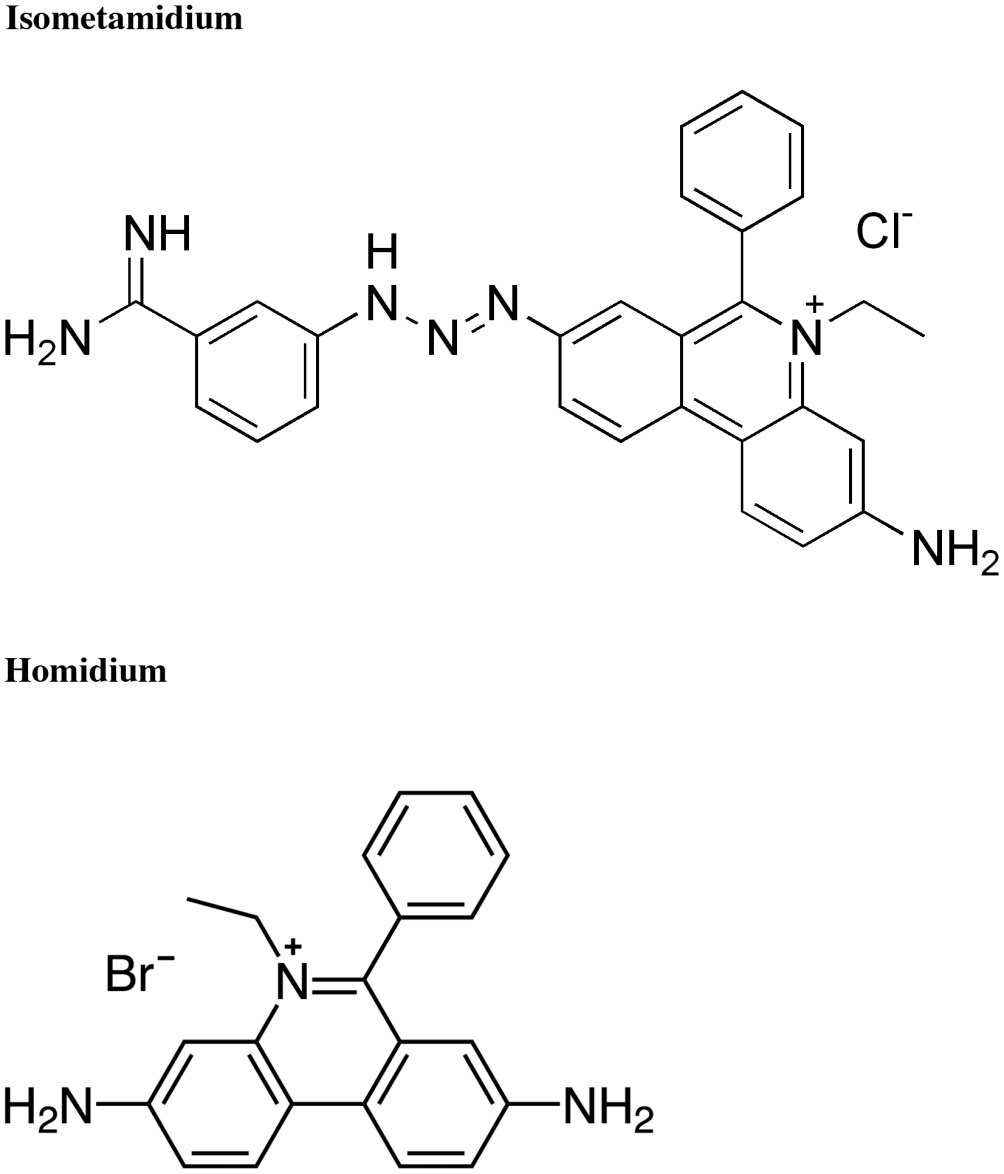
Structural relatedness between isometamidium and ethidium salts.

Homidium is part of the ISM molecule, the structural relatedness of both molecules being thus obvious (Figure 1). Furthermore, in the field, cross-resistance is observed between ethidium bromide and ISM [12] suggesting that uptake and extrusion of the drug within and from the trypanosome are mediated by the same mechanisms for both compounds.

Hence, our working hypothesis is that chemical compounds could interfere (compete) with the extrusion of ISM from the drug resistant trypanosome allowing a prolonged trypanocidal action. The objective of this work was to bring some indirect evidence confirming this working hypothesis. Preliminary experiments conducted *in vitro* would have allowed a more precise definition of the role of those secondary transporters in trypanocidal drug resistance but research in this domain is hampered by the fact that except for some atypical laboratory strains, bloodstreams forms of *T. congolense* do not grow properly in vitro [13]–[15]. Seeking a commercially available chemical compound that could be used for treating livestock, a number of antibiotics were selected and screened in a mouse model. The criterion for inclusion in this study was the affinity of the medications for bacterial efflux systems as described for β-lactams [16], [17], tetracycline (TC), oxytetracycline (OTC) [18], nalidixic acid (quinolone) [19] and the fluoroquinolone enrofloxacine (FQE) [20]. The nalidixic acid and β-lactam (Penicilline G) were rejected after preliminary experiments (see table 1), no difference being observed between groups treated with ISM alone or treated with ISM associated with one of the two compounds. The absence of curative effects of the antibiotics used alone was consistently checked in a mouse model (see table 1). After this preliminary screening, OTC was selected for the experiment in cattle as it is available as an injectable long acting form allowing for a reduction of the number of injections. For the experiment in mouse, TC was chosen as the easiest and cheapest commercial preparation for oral administration by dilution in drinking water. Enrofloxacin was not pre-tested in combination with ISM in mice but immediately used in cattle.

**Table 1:** Initial screening of antibiotics in a mouse model (*Trypanosoma congolense* strain IL3343).

Treatment	Cured	Median PP (days)	95% confidence intervals
Untreated control	0/6	5,41	4,10–7,16
Penicilline G (100mg/kg)	0/6	5,79	4,38–7,77
Nalidixic acid (175mg/kg)	0/6	6,29	4,76–8,32
Tetracycline (125mg/kg)	0/6	6,80	5,14–8,99
Oxytetracycline (125mg/kg)	0/6	5,06	3,83–6,69
Enrofloxacine (40mg/kg)	0/6	5,22	3,95–6,90
ISM (1mg/kg)	0/6	16,29	12,32–21,53
ISM/Penicilline G (*)	0/6	12,27	9,28–16,22
ISM/Nalidixic acid	0/6	16,73	12,66–22,13
ISM/Tetracycline	4/6	51.96	38.01–71.05
ISM/Oxytetracycline	3/6	39.1	29.16–52.43
ISM/Enrofloxacine	N.T.	-	-

(*) ISM at 1mg/kg combined to the antibiotic at the dose used alone; N.T.: Not Tested.

## Materials and Methods

### Ethics statement

This is to certify that the experiments carried out at the Institute of Tropical Medicine in the framework of the hereunder mentioned study were approved by the Ethics Committee of the Institute of Tropical Medicine and that the study was conducted adhering to the institutional guidelines for animal husbandry. In Belgium protection of experimental animals is regulated by the Royal Decision of 14/11/1993. Article 3bis paragraph 1 of this Royal Decision stipulates that: Every laboratory that keeps vertebrates with a view to perform experiments that may cause pain, suffering or lesions, has to establish an Ethics Committee. The Ethics Committee is composed of at least 6 members. The laboratory director or his representative, the leaders of the experiments, some laboratory assistants and the veterinary surgeon or the expert charged with the supervision of the health and the well-being of the animals are part of the Ethics Committee. Moreover one or more independent members, not belonging to the laboratory staff, will be member of the Committee. A veterinary inspector of the Ministry of Agriculture will also have a seat on the Ethics Committee. Identification of the experiment: DG008-VD-M-Tryp Title of the project: Study on the genetic basis and improved detection methods of resistance against isometamidium and diminazene in animal trypanosomes. Date of reception of the application: 03/11/2008 Date of approval by the Ethical Commission: 23/12/2008 (extension of a similar application DG006-VD-M-Tryp approved in 2004) Validity of this approval: from 23/12/2008 until 22/12/2012.

### Trypanosome strains

The cloned *T. congolense* savannah type strain IL3343 was identified as resistant to ISM when tested in mice (CD50 = 1.7 mg/kg) [21] with the CD50 defined as the curative dose that gives complete cure in 50% of the animals.

The *T. congolense* savannah type strain TRT57_C10_ was isolated from cattle in Eastern Zambia in 1996, cloned and conserved as a stabilate in liquid nitrogen. It was identified as highly resistant to ISM when tested in mice. Three doses of ISM, i.e. 0.1, 1 and 10mg/kg were tested according to the protocol described by Eisler et al. [22]. When treated with 10mg/kg ISM, 100% of the mice relapsed (CD50>10mg/kg).

### Mice inoculation and treatment

The stabilates of the cloned isolates were reactivated in mice. When the parasitaemia reached 8 on Herbert and Lumsden's scale [23], blood was collected under terminal anaesthesia by heart puncture and 4 groups of 16 adult OF1 mice weighing on average 30g each were inoculated with one of the two trypanosome clones (5*10^5^ trypanosomes/mouse through intraperitoneal injection). Twenty four hours after inoculation and for each clone, group 1 was left untreated and served as control, group 2 was treated for 30 days, per os, with 125mg/kg/day tetracycline, group 3 was treated with 1mg/kg ISM injected once intraperitoneally and finally, group 4 was treated with 1mg/kg ISM injected once intraperitoneally and was treated per os with 125mg/kg/day TC for 30 days. Mean water consumption of the mice was determined before and during the experiment and was on average of 3ml/day/mouse at 18°C. This water intake was not affected by the presence of TC in the drinking water. All mice were monitored three times a week for survival and presence of trypanosomes by microscopic examination of a wet film made from fresh blood sampled from the tail of each mouse for a period of 140 days.

Mice were euthanized when their health status, determined by clinical examination, was deteriorating (prostration, lateral decubitus, hyperventilation, unconsciousness and/or PCV≤20).

At day 140, all surviving mice were euthanized and between 1.5 and 2ml blood was collected. The DNA of the whole blood sample was then extracted using a routine phenol–chloroform–isoamyl alcohol method [24]. To confirm the presence or absence of trypanosomes, the PCR technique on the 18S small subunit of the ribosomal DNA (Ssu-rDNA) was used [25], [26].

### Cattle inoculation and treatment

5Three groups of 6 adult crossbred zebus weighing on average 158 kg each (extremes 140 and 201kg) were inoculated with 5×10^5^ trypanosomes (cloned isolate IL3343) each by intra-jugular injection 30 days after treatment with DA (7mg/kg) to clear all trypanosomal infections and deworming. One non-treated control group of 2 cattle was inoculated in the same way. The 20 cattle were housed in fly-proof facilities. From day 7 after the inoculation, all animals were monitored 2 times a week during 95 days. Their PCV was measured and jugular blood was examined for the presence of parasites by microscopic examination of the buffy coats and by PCR [25] performed on buffy coats collected on on Whatman 4 filter paper (Whatman). The DNA was obtained using a routine chelex-based extraction method [24].

At the first parasitaemia, group A was treated with one single administration of 0.5mg/kg ISM by intramuscular (IM) injection, group B with one single administration of 0.5mg/kg ISM and with 20mg/kg OTC (Terramycin LA) IM every 3 days for 30 days and group C with one single administration of 0.5mg/kg ISM and with 5mg/kg FQE (Baytril 100) IM every 2 days for 30 days. For each animal, the injection sites of the drugs were alternatively selected in forehand and hindquarters, shaved and coloured with methylene blue and picric acid for OTC and FQE respectively. A minimal distance of 6 cm between injection sites was respected.

### Statistical analysis

The survival of the mice in the 8 groups and of the cattle in the three groups was analysed in two separate survival models in Stata 10 (Copyright 1996–2009 StataCorp LP) using groups as an explanatory variable. A log-normal distribution was used in a parametric model (Text S1). The start of the model corresponded to the day of inoculation and the experiment was short enough to ignore natural mortality of animals.

The cattle's PCV values were analyzed using a cross-sectional linear regression, accounting for repeated measures from individual animals. Explanatory variables were the animal groups, post-treatment periods and the interactions between them. Three post-treatment periods each containing the same number of samplings were defined as follows: day 1–21, day 22–54 and day 55–98. The interaction term between the groups and the third period (using the first period as a baseline) was used as indicator of the impact of the disease on the PCV.

## Results and Discussion

### Experiment in mice

A significant effect of treatment and resistance to treatment on the survival time of the mice was observed. The data are summarized in table 2. Groups 3 and 4 survived significantly longer than group 1 (control without treatment; P<0.01), unlike group 2 (received only TC as treatment; P>0.1). The longer survival time of the mice treated with ISM with or without potentiator is confirming our field observations that even when trypanocidal drug resistance is present, ISM seems to impair the development of the parasite, reducing the impact of the disease on the health of the infected animal.

**Table 2:** Summarized data of the output of the treatments in mice.

	Group 1 (control)	Group 2 (TC)	Group 3 (ISM)	Group 4 (ISM-TC)
**TRT57C10**				
Number of animals	16	16	16	16
Median PP (days)	5,7 (4,2–7,8)	5,8 (4,3–7,9)	10,8 (8,0–14,8)	14,5 (10,6–19,7)
Median ST (days)	9,6 (7,9–11,8)	9,5 (7,8–11,6)	13,8 (11,3–16,9)	20,2 (16,5–24,7)
Cured	0	0	0	1
**IL3343**				
Number of animals	16	16	16	16
Median PP (days)	4,3 (3,2–5,9)	4,1 (3,0–5,6)	86.3 (61.4–121)	249 (153–405)
Median ST (days)	8,2 (6,7–10,0)	8,5 (7,0–10,4)	134 (106–172)	244 (169–355)
Cured	0	0	13	15

**Figure 2:**
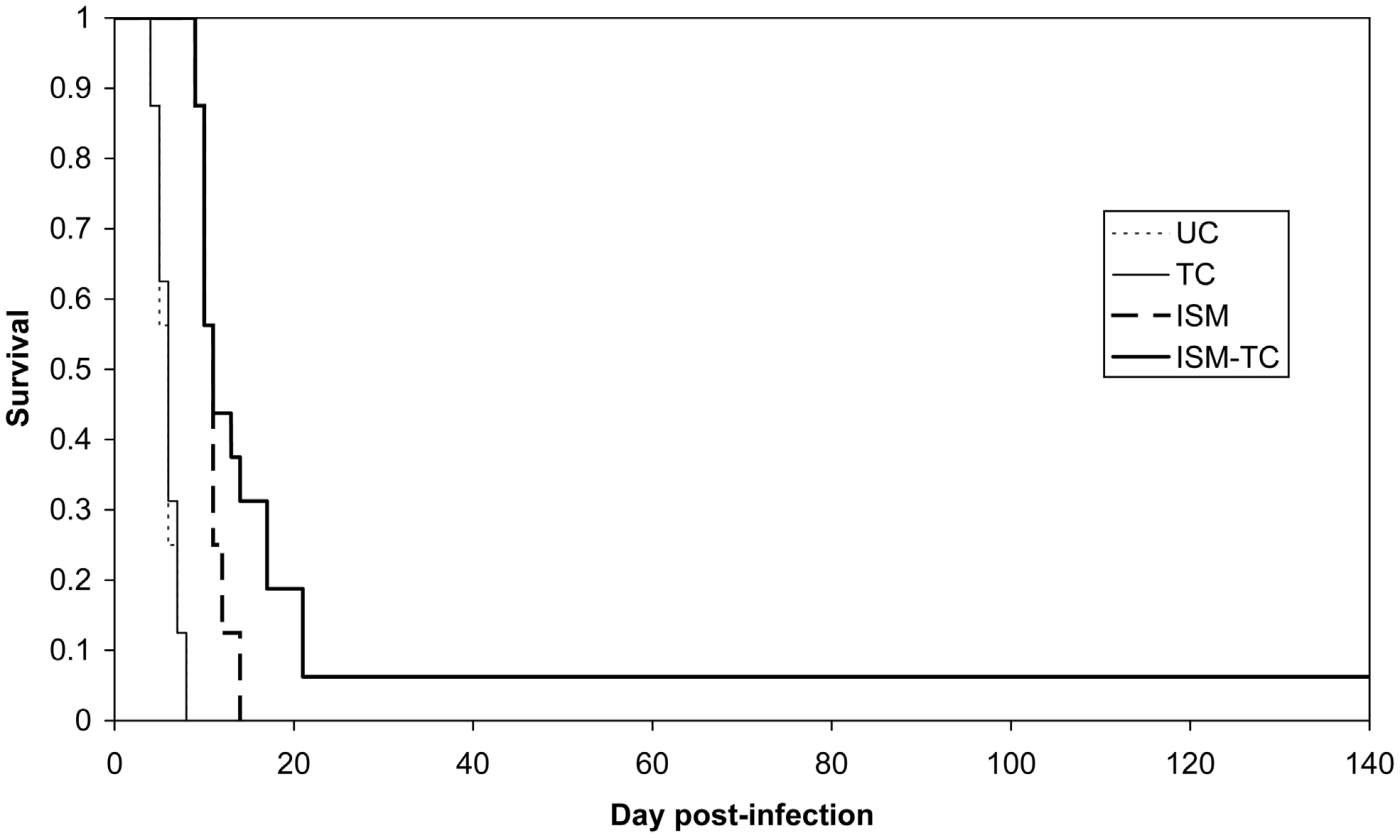
Kaplan-Meier survival estimates in mice infected with the highly resistant strain. UC: group 1 Untreated Control; TC: group 2; ISM: group 3; ISM-TC: group 4.

**Figure 3:**
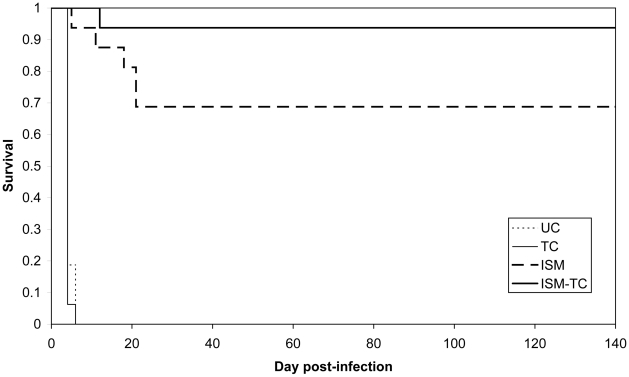
Kaplan-Meier survival estimates in mice infected with the resistant strain IL3343. UC: group 1 Untreated Control; TC: group 2; ISM: group 3; ISM-TC: group 4.

For both strains, resistant (IL3343) and highly resistant (TRT57_C10_), there was a significant difference between groups 3 (ISM treatment only) and 4 (treated with ISM and TC; P<0.01) (Figure 2 and Figure 3). When considering the efficacy of the compounds against the trypanosomes, the complete ineffectiveness of TC alone and the increased efficacy of ISM in presence of TC, provides strong arguments in favor of the hypothesis that the two compounds compete for the same efflux system.

Despite the unusual high degree of resistance of *T. congolense* TRT57_C10_, the survival times were significantly higher after treatment with the association of ISM and TC. One mouse survived the infection for 140 days. Such a high survival time was never observed before in laboratory experiments using this strain. Furthermore, the PCR analysis of the blood sample at day 140 was negative suggesting that the trypanosomal infection was cleared completely. Moreover, the blood sample used for diagnosis was between 1.5 and 2ml of blood representing the average total amount of blood that can be collected from a mouse. Since the sensitivity of the diagnostic method is 25 trypanosomes/ml [25], the complete absence of trypanosomes and thus, the complete clearance of the parasites from the host can reasonably be assumed.

For the resistant strain (IL3343), in group 3, among the 13 surviving mice, 3/13 were microscopically positive for the presence of trypanosomes and 6/13 were positive for the presence of trypanosomes by PCR. In group 4, among the 15 surviving mice, 1/15 was microscopically positive for the presence of trypanosomes and 3/15 were positive for the presence of trypanosomes by PCR.

### Experiment in cattle

The two untreated control animals became parasitaemic 11 days after inoculation and were treated with DA (7 mg/kg) on day 30 because their PCV reached the critical value of 25.

**Figure 4:**
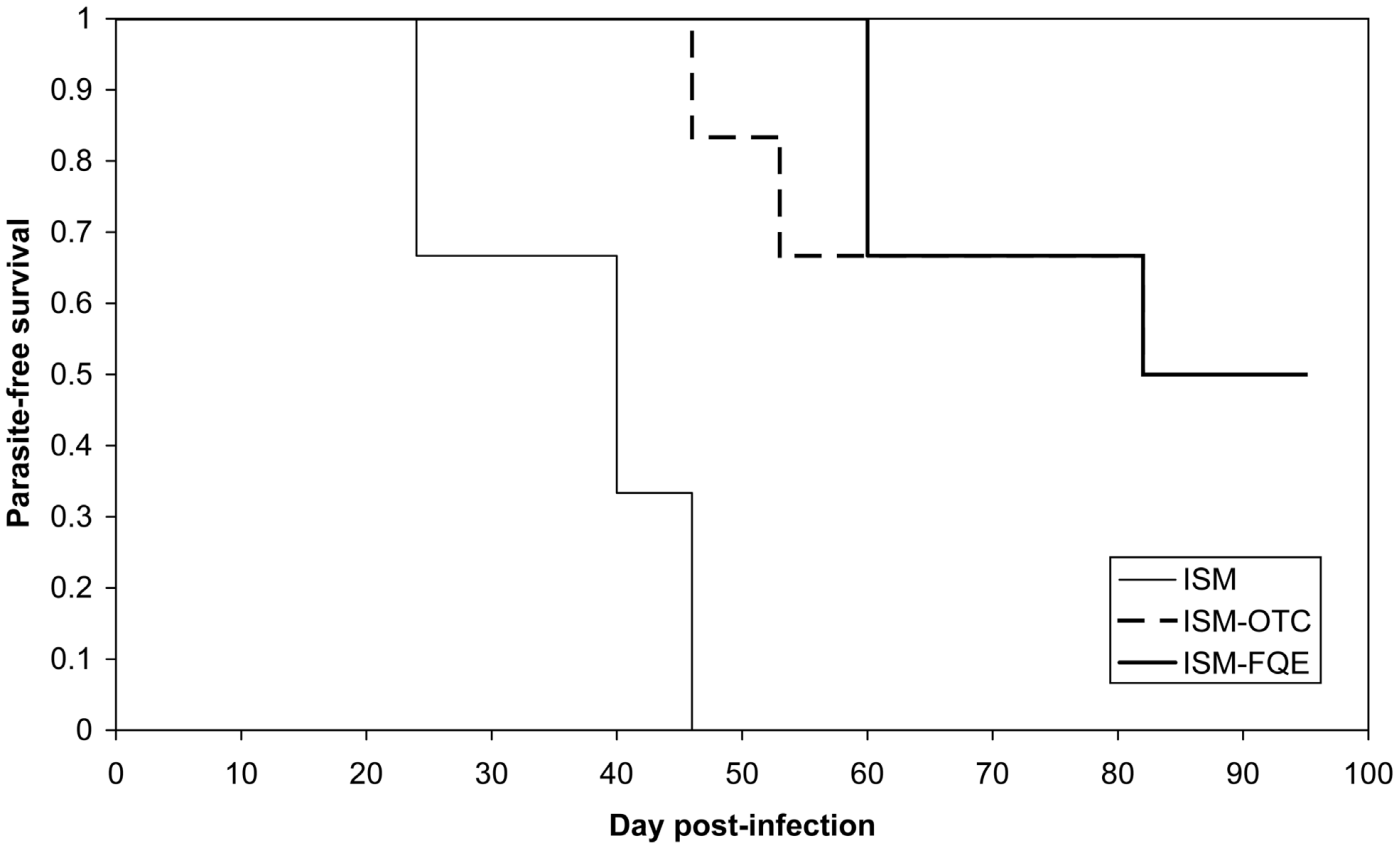
Kaplan-Meier survival estimates in cattle infected with the resistant strain IL3343. ISM: group A; ISM-OTC: group B; ISM-FQE: group C.

All 6 animals of group A (ISM) became positive between days 24 and 46 post-inoculation. The data are summarized in table 3. When ISM was used in combination with either OTC (group B) or FQE (group C), the prepatent period was significantly longer (p<0.001; Figure 4). 50% of the cattle became infected (between days 46 and 82) and 50% completely cleared the infection. In the groups B (ISM-OTC) and C (ISM-FQE), the parasitaemia remained very low, below the detection level of the microscopic examination, i.e. 450 trypanosomes/ml [27]. The PCR results were fluctuating with animals being detected parasitaemic every 2 to 3 weeks, indicating a parasitaemia oscillating just above and below the detection limit of the PCR test, i.e. 25 trypanosomes/ml blood [25].

**Table 3:** Summarized data of the output of the treatments in cattle.

	Group A (ISM)	Group B (ISM-OTC)	Group C (ISM-FQE)
**Number of animals**	6	6	6
**Median prepatent period (days)**	35 (26–47)	84 (61–117)	91 (66–127)
**Mean PCV drop from period 1 to period 3**	5.9 (4.5–7.3)	1.8 (0.3–3.2)	3.3 (1.9–4.7)
**Cured**	0	3	3

The impact of the infection on the PCV was not very pronounced, even in group A (average PCV reduction 8 to 14 weeks after treatment: 5.9%; 95% CI: 4.5–7.3). However, this impact was lower in groups B (ISM-OTC) and C (ISM-FQE) compared to group A (ISM) (p<0.01). These observations indicate that even in the case of ISM-resistant trypanosomes, farmers still seem to benefit from the use of the trypanocide because of the significant decrease of the effect of the infection on the health status of the animals as represented in the PCV values.

### Prospects and impact

Although resistance to DA and ISM, is developing quickly [5], [28], [29], controlling the parasite in livestock using drugs remains the control method of choice for small-scale livestock breeders. Localised tsetse control is usually not effective [30] and a vaccine is not yet available, leaving little choice to control the disease. Trypanosomiasis not only affects livestock production (milk, meat) but also impacts greatly on crop production through the inability to keep draft animals in tsetse-infested areas [31]. Notwithstanding the alarming levels of trypanocidal drug resistance that have been reported in the cotton belt of West Africa [32] and in some regions of southern Africa (including Zambia) [4], [5], new trypanocidal drugs for animal use are not expected to become available in the near future. Pharmaceutical companies do not invest in research and development of new veterinarian trypanocidal compounds for a too specific, limited African market with poor benefit perspectives [33]. Hence, potentiating the available trypanocidal drugs may represent a powerful alternative to the current problems associated with the control of trypanosomes in livestock. Research in the field of non-competitive inhibitors of efflux pumps in bacteria is being conducted [34]–[36] and may ultimately represent an immense hope for future control of trypanosomiasis using drugs. In the meantime, TC and some derivatives are cheap drugs, registered for use in livestock, widely available on the African market and with an expired patent, now in the public domain. More importantly, TC is commonly used by African farmers and will not require elaborate new chemistry and safety tests. Hence, assuming that further trials confirm the effectiveness of the antibiotics in potentiating the activity of trypanocidal drugs in cattle under natural tsetse challenge, the new control approach can be implemented rapidly. It is likely that the combination ISM–TC/OTC can also be made more cost effective after adjusting dosage and the duration of the treatment. Furthermore, several analogues of TC/OTC and FQE are available albeit somewhat more expensive as patents are still in force. These compounds are currently being screened with the aim of optimizing the delivery system to increase the specificity of the treatment, to boost the intracellular concentration of the chemosensitizer within the trypanosome and to reduce the dose. Obviously, the current treatment schedule cannot be used under field conditions. The repeated administration of a high dose of antibiotics is far too expensive for the rural communities and would certainly render the treated animals unsuitable for human consumption. Further research is thus ongoing to identify the best galenic solution, the optimal combination of chemosensitizer with ISM (qualitative and quantitative) and to test this combination in livestock under controlled and field conditions in areas with high tsetse challenge and high trypanocidal drug resistance. An effective combination of ISM and chemosensitizer(s) should result in (i) a decrease in the proportion of circulating strains resistant to ISM and (ii) a decrease in the impact of the disease on the health status of the cattle. Strategic use of this approach may result in an increased efficacy of currently available trypanocidal drugs in extensive areas of sub-Saharan Africa where their use is severely curtailed as a result of the development of resistance in trypanosomes.

## Supporting Information

Text S1 [Statistics] Model of the overall hazard as a function of time using an exponential mode.
(0.02 MB DOC)
