# Supplementary material for: Chemosensitization of Trypanosoma congolense Strains Resistant to Isometamidium Chloride by Tetracyclines and Enrofloxacin
Source: PLoS Negl Trop Dis. 2010 Sep 28;4(9):e828. doi: 10.1371/journal.pntd.0000828 (PMC2946901; doi:10.1371/journal.pntd.0000828)
Supplement: Text S1 — [Statistics] Model of the overall hazard as a function of time using an exponential mode. (0.02 MB DOC) [file pntd.0000828.s001.doc]

To investigate the appropriateness of the log-normal distribution, the overall hazard was modelled as a function of time, using an exponential model. The dataset was split to generate data for five periods (t_1_-t_5_) containing approximately each the same number of events. Including this new time variable in an exponential model gives the following function: |

$$h_{(t\text{|x)}}=h_{0}.e^{(b.x+c.t)}=h_{0}.e^{(c.t)}.e^{(b.x)}$$

where:

*h_(t|x)_* is the hazard rate at a given time and in a given group

*h_0_* is the baseline hazard rate

*b* and *c* are the coefficients of, respectively, the survival model’s explanatory variables (*x*) and the time period (*t*)

In this equation, $h_{0}.e^{(c.t)}$ is the baseline hazard rate in a given period. The hazard rate is defined as the average number of failures/events (lethal parasitaemia in this case) observed in one individual exposed to the risk for one unit of time (one day in this case).

Log-normal distributions are particularly well adapted to situations as in this experiment, where the risk of failure (lethal parasitaemia) exhibits a mode in function of the time (the risk is very low at the beginning of the experiment, reaches a peak after a few days and decreases again).The results of the the baseline hazard split by period of time confirm that the use of the log-normal distribution is appropriate for the data. The first days following the infection, the probability that an infected animal dies is very low. Afterwards, the hazard rate increases exponentially until it reaches a peak value and decreasing at a slower rate.
